# Investigation of Drug-Interaction Potential for Arthritis Dietary Supplements: Chondroitin Sulfate, Glucosamine, and Methylsulfonylmethane

**DOI:** 10.3390/molecules28248068

**Published:** 2023-12-13

**Authors:** Su Min Kim, So Young Jo, Ho-Young Park, Yu Ra Lee, Jun Sang Yu, Hye Hyun Yoo

**Affiliations:** 1 Institute of Pharmaceutical Science and Technology, College of Pharmacy, Hanyang University, Ansan 15588, Republic of Korea; ksm990201@naver.com (S.M.K.); sjo7909@gmail.com (S.Y.J.); 2 Food Functionality Research Division, Korea Food Research Institute, Wanju-gun 55365, Republic of Korea; hypark@kfri.re.kr (H.-Y.P.); lyr@kfri.re.kr (Y.R.L.)

**Keywords:** osteoarthritis, chondroitin sulfate, glucosamine, methylsulfonylmethane, cytochrome P450

## Abstract

Osteoarthritis is one of the leading conditions that promote the consumption of these dietary supplements. Chondroitin sulfate, glucosamine, and methylsulfonylmethane are among the prominent alternative treatments for osteoarthritis. In this study, these dietary supplements were incubated with cytochrome P450 isozyme-specific substrates in human liver microsomes, and the formation of marker metabolites was measured to investigate their inhibitory potential on cytochrome P450 enzyme activities. The results revealed no significant inhibitory effects on seven CYPs, consistent with established related research data. Therefore, these substances are anticipated to have a low potential for cytochrome P450-mediated drug interactions with osteoarthritis medications that are likely to be co-administered. However, given the previous reports of interaction cases involving glucosamine, caution is advised regarding dietary supplement–drug interactions.

## 1. Introduction

Osteoarthritis (OA) is a condition characterized by the gradual deterioration of joint tissues as time progresses [1]. It stands as the most prevalent type of arthritis, impacting approximately 12% of the population [2]. The increasing prevalence of OA, which leads to disability in elders aged 60 years or older, can be attributed to the aging demographic and escalating obesity rates [3]. The guidelines for OA treatments are summarized in Table S1 [4]. While variations exist across guidelines, commonly prescribed oral medications for OA include acetaminophen, nonsteroidal anti-inflammatory drugs (NSAIDs), opioids, and systemic slow-acting drugs in osteoarthritis (SYSADOA). Intra-articular injections can be considered. However, several studies have indicated issues associated with these medications, such as acetaminophen, NSAIDs, and opioids [5,6]. Therefore, public interest in the benefits of alternative treatments, including SYSADOA, has increased. Chondroitin sulfate (CS), glucosamine (GCS), and methylsulfonylmethane (MSM) belong to the category of SYSADOA, with varying recommendations or contraindications in different guidelines [4]. Nevertheless, these substances are often used indiscriminately, irrespective of guideline recommendations, raising the potential for concurrent use with other medications.

CS is a glycosaminoglycan consisting of a polymerized disaccharide base linked to a sulfate group. It is typically present in the proteoglycans found in articular cartilage. In dietary supplements, CS is commonly sourced from bovine trachea, although alternative sources such as ovine or porcine trachea and shark skeletons (shark cartilage) are also utilized in some dietary supplements [7]. GCS is an amino sugar precursor, serving a pivotal role in the formation of glycosylated proteins and lipids. GCS is one of the most abundant monosaccharides in the human body. It has been observed to potentially enhance aggrecan synthesis, reduce inflammation by controlling protease catabolic activity, and manifest a variety of positive effects. Additionally, it is established that GCS may stimulate the production of hyaluronic acid in the synovial membrane while inhibiting cartilage activity through the degradation of liposomal enzymes [8]. MSM is the oxidized cartilage shown to be one-third the level of dimethylsulfoxide (DMSO), a natural, organic form of sulfur. It is a much more stable organic sulfur compound with medicinal properties equal to those of DMSO but without the odor and skin irritation complications of the latter. MSM is an effective natural analgesic. It blocks the inflammatory process and enhances the activity of cortisol, a natural anti-inflammatory hormone produced in the body [9]. The majority of OA patients are elderly, and a significant portion (69% of the entire patient population) use dietary supplements as alternative therapies [2]. Therefore, there is a high likelihood of concomitant use with analgesics and NSAIDs employed for OA, as well as medications for age-related and chronic diseases, causing dietary supplement–drug interactions.

The primary catalysts involved in such interactions are cytochrome P450 (CYP) enzymes. CYP enzymes are metabolic enzymes engaged in over 90% of documented enzymatic processes. CYP plays a vital role in drug metabolism, cellular processes, and equilibrium, exerting a profound influence on the efficacy and safety of medications. Notably, many drugs, foods, and substances can induce or inhibit CYP, leading to drug interactions that may result in unexpected adverse effects or treatment ineffectiveness [10]. To the best of our knowledge, there is a lack of data evaluating CYP inhibition of GCS, CS, and MSM in human liver microsomes to predict CYP-mediated drug interactions. Therefore, this study aims to assess the CYP inhibitory effects of GCS, CS, and MSM with human liver microsomes using the LC-MS/MS cocktail method.

## 2. Results

### 2.1. CYP Inhibition Assay

**Figure 1:**
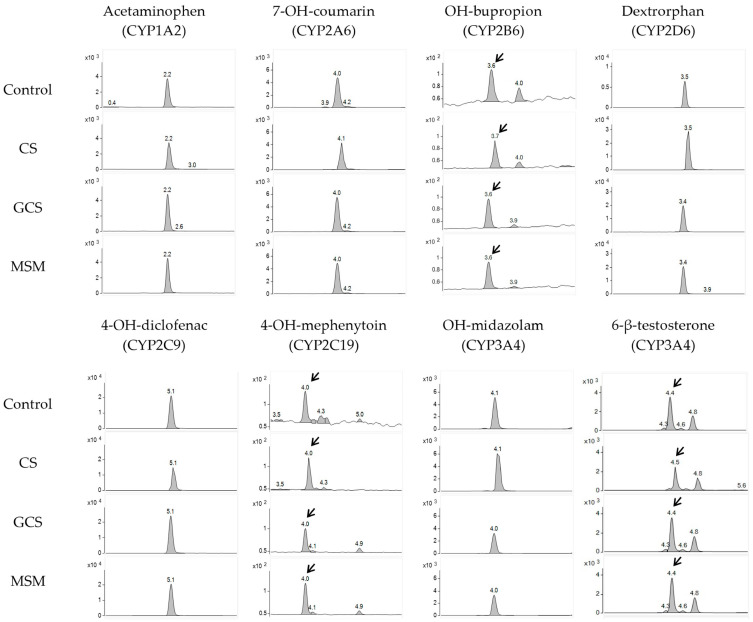
Representative analysis chromatograms of probe metabolites in control and CS (100 μM), GCS (300 μM), and MSM (300 μM)-treated samples. Arrows show peak of probe metabolites.

In this study, the inhibitory effects of CS, MSM, and GCS on seven different CYP isozymes were investigated using human liver microsomes. To validate the experimental conditions, positive controls were performed using potent selective inhibitors for each CYP isozyme, resulting in a significant reduction (>90%) in the formation of CYP-specific metabolites, thus, confirming the reliability of the experimental and analytical conditions. Subsequently, the inhibitory effects of CS, MSM, and GCS on seven different CYP isozymes were evaluated at various concentrations, and the peak area values of metabolites for each CYP enzyme were compared with the control group. The inhibition assay results of CS, MSM, and GCS are presented in Table 1, Table 2, and Table 3, respectively. The chromatograms of the samples are shown in Figure 1.

At every concentration tested, the peak area values of metabolites produced by human liver microsomes remained above approximately 80% when compared to the control group, indicating that these three test compounds have no inhibitory effects on the seven tested CYP isozymes. As the concentration of test compounds increased, there were no significant reductions in the area values, indicating that the IC_50_ for CYP inhibition was expected to be above 1000 µM, and the CYP inhibition effects of the three substances were negligible.

### 2.2. Method Validation

#### 2.2.1. Linearity

The results and data are presented in Figure S1 and Table S2. The calibration curves established for the probe metabolites were validated for the analysis of metabolites within human microsomal incubations. Triplicate standards were employed at various concentrations to generate these curves. Good linearity was observed, as indicated by correlation coefficients exceeding 0.99 across all probe metabolites. The resulting accuracy and precision values fell within the acceptable range of ±15.0%.

#### 2.2.2. Selectivity

The results and data are presented in Figure S2. The selectivity of the method was evaluated by comparing it with blank, control, and spiked samples to determine the degree of chromatographic interference from the matrix background. It was confirmed that there were no other interference peaks in the blank samples. Other impurities and probe metabolites in the control sample appeared well separated.

#### 2.2.3. Accuracy and Precision

The results and data are presented in Table S3. The accuracy and precision of the method were assessed by analyzing three replicates of quality control samples at concentrations of 6%, 30%, and 70% of the set concentrations of probe metabolites within a day and over three different days. Accuracy was assessed by comparing the measured concentrations with the theoretical concentrations (%), while precision was evaluated through the relative standard deviation (RSD, %). For eight probe metabolites, the intra-day accuracy varied from 88.12% to 106.15%, and precision ranged from 0.07% to 10.98%. In the inter-day assessment, accuracy values varied from 89.22% to 104.29%, with precision found to be between 0.07% and 4.31%. For both intra-day and inter-day assessments, accuracy and precision were confirmed to be within the acceptable limit of ±15% and 15%, as specified by the FDA bioanalysis method validation guidelines.

## 3. Discussion

In this study, none of the three substances, CS, MSM, and GCS, exhibited significant inhibitory effects on seven different CYPs. Previous pharmacokinetic studies for each substance, at common daily doses of 1200 mg/day for CS, 1500 mg/day for GCS, and 2000 mg/day for MSM, reported maximum plasma concentrations of approximately 70, 9, and 1100 µM, respectively [11,12,13]. Therefore, given that the concentrations tested in this study were either higher or similar to those achieved at daily doses, it is inferred that these substances are unlikely to exhibit CYP-mediated drug interactions at their respective daily doses. These results were supported by previous studies that found no inhibitory effects of CS and GCS on CYP2D6 and CYP2C9 [14,15]. Furthermore, through in vivo mice, intrahepatic gene expressions of CYPs were evaluated after the administration of MSM, and there were no significant inhibition or induction effects on CYPs [16].

According to the types of drugs used for OA, representative medications were selected, and information regarding related CYPs and CYP-mediated drug interactions is presented in Table 4. Acetaminophen, tramadol, codeine, and pethidine were chosen among the analgesics for OA. Co-administration of acetaminophen with CYP inducers such as isoniazid, carbamazepine, and rifampicin has been reported to increase hepatotoxicity due to more formations of toxic metabolites [17,18]. In the case of tramadol, concomitant use with the CYP2D6 inhibitors, escitalopram and terbinafine, led to an increase in plasma concentrations of tramadol, while co-administration with the CYP inducer rifampicin resulted in decreased plasma levels of tramadol [19,20,21]. For codeine, a controlled substance, it has been challenging to conduct clinical trials with CYP inhibitors. However, a study involving post-mortem examinations related to fatal cases of codeine use revealed a significant reduction in the codeine-to-metabolite ratio depending on the presence of CYP2D6 inhibitors [22]. As for pethidine, concurrent use with the CYP inducer phenobarbitone increased metabolic activity, while co-administration with the CYP inhibitor chlorpromazine reduced metabolic activity [23].

Among NSAIDs, four drugs were selected: ibuprofen, diclofenac, meloxicam, and celecoxib. These NSAIDs were primarily metabolized by CYP2C9. Ibuprofen displays stereoselectivity, with the R-enantiomer primarily metabolized by CYP2C8 and the S-enantiomer by CYP2C9. Inhibitors of CYP2C9, including voriconazole and fluconazole, increased the plasma concentration of S-ibuprofen, while the CYP2C8 inhibitor gemfibrozil leads to an increase in the plasma concentration of R-ibuprofen [28,29]. For diclofenac, co-administration with the CYP2C9 inhibitor voriconazole increases the plasma concentration of diclofenac [30]. Furthermore, co-administration of meloxicam with the CYP2C8 inhibitor amiodarone and voriconazole increases the plasma concentration of meloxicam, while co-administration of meloxicam with the CYP3A4 inhibitor itraconazole decreases the plasma concentration of meloxicam [33,34]. The mechanism of interaction between itraconazole and meloxicam is anticipated to involve factors other than direct inhibition of CYP3A4, and it has not yet been elucidated. Co-administration of celecoxib with CYP2C9 inhibitors, fluconazole and fluvastatin, increases the plasma concentration of celecoxib, and with the CYP2C9 inducer rifampicin, it leads to a decrease in plasma concentration [36]. Therefore, when combined with CYPs inhibitors or inducers with commonly used analgesics and NSAIDs for OA, there is potential for modulating plasma concentrations and metabolite formations, causing adverse events. In this study, CS, GCS, and MSM did not demonstrate inhibitory effects on all CYP enzymes tested, suggesting that they can be considered safe concerning CYP-mediated drug interactions.

Generally, clinical trial results suggest that CS, GCS, and MSM are safe substances when taken at typical doses as monotherapy, and do not exhibit significant adverse effects [7,37,38]. However, there have been reports of an interaction case where the co-administration of CS and GCS with warfarin resulted in an elevated INR [39]. In addition, a scientific opinion by the European Food Safety Authority (EFSA) has indicated the potential for drug interactions leading to INR elevation in 40 cases when coumarin anticoagulants were combined with GCS [40]. Furthermore, a study demonstrated an increase in paracetamol’s AUC and maximum plasma concentration when co-administered with GCS in a 1:4 (paracetamol:GCS) ratio in rats, suggesting a potential metabolic interaction via an inhibitory effect on CYP2E1 [41]. Paracetamol, also known as acetaminophen, is one of the primary medications used for OA, and it has a high likelihood of concomitant use with GCS. Additionally, it interacts with various drugs, including alcohol, and can lead to hepatotoxicity. As a result, it is a drug with strict daily usage limitations [17]. Although this is an in vivo study conducted in rats, if it operates similarly in humans and leads to an increase in the plasma concentration of paracetamol, there is potential for hepatotoxicity [41]. However, the paper proposed CYP2E1 inhibition as the mechanism, but a related report has indicated no inhibitory effect of GCS on CYP2E1 in another study [1]. Therefore, further relevant research is warranted. Based on the results of this study, it is anticipated that CS, GCS, and MSM’s catalytic inhibition effects on the seven CYPs are negligible, indicating no significant interactions when co-administered with other medications. Nonetheless, given the existence of interaction cases, caution of dietary supplement–drug interactions should be advised.

CYP polymorphism significantly impacts drug metabolism and stands as a crucial factor in drug interactions [42,43]. Although this study did not reveal any inhibitory effect of the three substances on CYP, it is essential to exercise caution due to potential variations in metabolic activity based on polymorphism. Therefore, careful attention is warranted, considering the potential impact of polymorphism on metabolic activity, despite the absence of observed inhibitory effects in this study.

## 4. Materials and Methods

### 4.1. Chemicals and Materials

Chondroitin sulfate was purchased from GlpBio (Montclair, CA, USA), dimethyl sulfone (methylsulfonylmethane) was purchased from Toronto Research Chemicals (North York, ON, Canada), and glucosamine hydrochloride was purchased from Sigma-Aldrich Co. (St. Louis, MO, USA). Pooled human liver microsomes were purchased from BD Gentest (Franklin Lakes, NJ, USA). Glucose 6-phosphate (>98%), β-NADP^+^ (>95%), glucose 6-phosphate dehydrogenase, phenacetin (>98%), acetaminophen (>99%), coumarin (>99%), 7-OH-coumarin (>98%), bupropion (>98%), (±)-hydroxybupropion (>98%), diclofenac (>98.5%), 4′-OH-diclofenac (>98%), mephenytoin (>98%), 4′-OH-mephenytoin (>98%), dextromethorphan (>98%), dextrorphan (>99%), midazolam (>98%), 1′-OH-midazolam (>98%), testosterone (>99%), 6′-OH testosterone (>98%), furafylline (>98%), 8-methoxsalen (>98%), quercetin (>95%), sulfaphenazole (>98%), ticlopidine (>99%), quinidine (>98%), and ketoconazole (>99%) were purchased from Sigma-Aldrich Co. All other chemicals were obtained at analytical grade and used without further purification. Distilled water was prepared using a Milli-Q purification system (Millipore, Burlington, MA, USA). All standard solutions and mobile phases were passed through a 0.22 µm membrane filter before use. CS and MSM stock solutions were prepared in distilled water (DW) at 40 and 200 mM, respectively. The stock solution of GCS was prepared in DMSO at a concentration of 200 mM.

### 4.2. Microsomal Incubation

For the experimental method, the reference method was used [44]. Briefly, incubation mixtures were composed of 0.5 mg/mL human liver microsomes; varying concentrations of CS in DW (0.1, 0.3, 1, 3, 10, 30, 100, and 200 µM) or MSM in DW (0.1, 0.3, 1, 3, 10, 30, 100, 300, and 1000 µM) or GCS in DMSO (0.1, 0.3, 1, 3, 10, 30, 100, 300, and 1000 µM); a substrate mixture in DMSO (40 µM phenacetin for CYP1A2; 2.5 µM coumarin for CYP2A6; 80 µM bupropion for CYP2B6; 10 µM diclofenac for CYP2C9; 80 µM [±]-mephenytoin for CYP2C19; 5 µM dextromethorphan for CYP2D6; 2.5 µM midazolam and 30 µM testosterone for CYP3A4); and an NADPH generating system (NGS; 0.1 M glucose-6-phosphate, 10 mg/mL β-NADP^+^, and 1 U/mL glucose-6-phosphate dehydrogenase) in a total volume of 200 µL potassium phosphate buffer (0.1 M, pH 7.4). The reaction mixture, excluding NGS, underwent pre-incubation at 37 °C for 5 min, followed by further incubation with NGS for 30 min in a water bath. Well-established selective CYP inhibitors were employed as positive controls (40 µM ketoconazole for CYP3A4, 50 µM furafyllin for CYP1A2, 10 µM quinidine for CYP2D6, 125 µM ticlopidine for CYP2C19 and CYP2B6, 10 µM 8-methoxsalen for CYP2A6, and 10 µM sulfaphenazole for CYP2C9). Post incubation, the reaction was halted by adding 50 µL ice-cold 1% formic acid acetonitrile. After centrifugation at 13,200 rpm for 5 min, the supernatant was collected from the sample and subjected to analysis via an LC-MS/MS system. All experiments were performed in triplicate.

### 4.3. LC-MS/MS Analysis

For the experimental method, the reference method was used [45]. The LC-MS/MS system utilized in this study consisted of an Agilent 1260 binary pump HPLC system coupled with the Agilent 6460 Triple Quadrupole mass spectrometer (Agilent Technologies, Santa Clara, CA, USA), featuring an electrospray ionization source. Chromatographic separation was achieved using a Fortis C8 column (2.1 × 100 mm, 5.0 µm; Fortis Technologies Ltd., Neston, UK). The HPLC mobile phases comprised (A) 0.1% formic acid and (B) 0.1% formic acid in 90% acetonitrile. A gradient elution method was employed with an initial solvent B concentration of 15% and a flow rate of 0.25 mL/min. The composition of solvent B changed as follows: 0–3.0 min, gradually increased to 85%; 3.0–4.5 min, maintained at 85%; 4.5–4.6 min, decreased to 15%; 4.6–11.0 min, re-equilibrated at 15%, for 6.4 min. The total run time was 11.0 min, and the injection volume was 5 µL. Mass detection was conducted in the positive ion mode using multiple reaction monitoring (MRM). Specific MRM transitions for each analyte are detailed in Table S4.

### 4.4. Method Validation

For the validation method, the reference method was used [46]. Selectivity, linearity, accuracy, and precision were validated according to the criteria in the FDA guidance, and the method validation was performed as mentioned in Section 4.2 and Section 4.3. In the blank sample, DMSO was spiked instead of the inhibitors and substrates. Linearity was assessed by spiking the sample in the blank sample with the following concentrations: 2, 5, 10, 20, 50, and 100% based on the area of each control sample (20 µM acetaminophen for CYP1A2; 10 µM 7-OH-coumarin for CYP2A6; 80 µM OH-bupropion for CYP2B6; 10 µM 4-OH-diclofenac for CYP2C9; 4 µM 4-OH-mephenytoin for CYP2C19; 1 µM dextrorphan for CYP2D6; 3 µM 1-OH-midazolam and 30 µM 6- β-OH-Testosterone for CYP3A4 were 100% concentrations, respectively). Quality control samples were low, medium, high-quality control (LQC, MQC, HQC) with 6, 30, 70% of setting concentrations, respectively. Intra-day accuracy and precision were evaluated with triplicates, and the inter-day assessment was conducted over a three-day period, with each experiment executed in triplicate.

## 5. Conclusions

In this study, the pharmacokinetic drug-interaction potentials of arthritis dietary supplements, CS, GCS, and MSM, were evaluated through the CYP inhibition assay. As a result, CS, GCS, and MSM had no inhibitory effects on CYP1A2, CYP2A6, CYP2B6, CYP2C9, CYP2C19, CYP2D6, and CYP3A4. These results suggest that CS, GCS, and MSM have a low likelihood of exhibiting CYP-mediated drug interactions with other medications. Despite these results, several cases of drug interaction involving CS and GCS have been reported. Therefore, additional research and careful consideration are advised regarding their potential interactions.

## Figures and Tables

**Table 1:** Effects of CS on CYP-specific metabolite formation in human liver microsomes (*n* = 3).

CYP Isozyme	Remaining Activities (% of Control, *n* = 3)							
	CS (μM)							
	0.1	0.3	1	3	10	30	100	200
CYP1A2	99.5 ± 4.0	96.9 ± 10.5	95.1 ± 13.3	106.8 ± 4.1	109.6 ± 3.7	108.6 ± 2.3	113.4 ± 16.5	100.3 ± 4.9
CYP2A6	101.2 ± 0.9	99.8 ± 7.1	99.5 ± 10.7	109.6 ± 1.8	111.1 ± 0.7	116.6 ± 4.6	124.9 ± 9.4	116.4 ± 1.4
CYP2B6	94.9 ± 3.9	99.0 ± 3.3	89.7 ± 14.4	99.1 ± 1.9	99.0 ± 6.2	100.6 ± 8.6	100.1 ± 8.0	106.4 ± 9.3
CYP2C9	105.3 ± 2.7	105.7 ± 2.5	102.4 ± 11.2	111.1 ± 1.0	115.6 ± 4.7	117.3 ± 4.2	121.3 ± 9.2	117.3 ± 2.4
CYP2C19	88.4 ± 6.2	90.6 ± 7.0	86.7 ± 9.6	94.7 ± 8.3	98.2 ± 10.2	106.0 ± 5.9	112.7 ± 12.6	119.9 ± 17.0
CYP2D6	100.5 ± 1.4	102.2 ± 5.8	99.0 ± 9.6	100.8 ± 1.1	105.7 ± 4.4	103.0 ± 3.7	104.4 ± 8.6	103.5 ± 6.4
CYP3A4(M) *	97.6 ± 3.8	99.1 ± 3.1	91.2 ± 10.6	87.9 ± 27.9	101.4 ± 4.9	106.6 ± 5	113.6 ± 8.4	114.6 ± 2.8
CYP3A4(T) *	101.7 ± 1.4	96.9 ± 7.0	97.8 ± 6.1	100.9 ± 0.6	107.5 ± 3.2	102.9 ± 0.7	109.3 ± 7.7	98.8 ± 2.6

* CYP3A4(M)-1-OH-midazolam; CYP3A4(T)–6-β-OH-Testosterone.

**Table 2:** Effects of MSM on CYP-specific metabolite formation in human liver microsomes (*n* = 3).

CYP Isozyme	Remaining Activities (% of Control, *n* = 3)							
	MSM (μM)							
	0.3	1	3	10	30	100	300	1000
CYP1A2	95.5 ± 12.9	103.7 ± 3.5	98.7 ± 5.1	103.0 ± 5.8	99.8 ± 6.6	101.8 ± 9.0	104.2 ± 7.5	101.2 ± 7.3
CYP2A6	98.7 ± 7.7	103.1 ± 3.3	101.1 ± 4.3	103.5 ± 5.1	102.1 ± 3.3	102.5 ± 5.0	103.5 ± 4.1	100.8 ± 3.6
CYP2B6	98.9 ± 8.0	104.9 ± 4.2	103.3 ± 6.0	104.3 ± 4.3	106.0 ± 4.1	105.00 ± 1.3	108.5 ± 7.5	106.9 ± 6.0
CYP2C9	106.7 ± 10.0	111.4 ± 3.4	108.1 ± 3.5	111.6 ± 6.8	110.5 ± 4.3	109.8 ± 4.3	110.8 ± 4.3	109.3 ± 6.1
CYP2C19	101.1 ± 11.0	108.2 ± 0.9	101.5 ± 9.2	105.7 ± 6.4	102.1 ± 7.1	102.7 ± 8.1	106.3 ± 9.5	102.6 ± 11.6
CYP2D6	96.8 ± 6.3	100.9 ± 1.6	98.0 ± 3.4	100.4 ± 2.5	98.5 ± 3.6	98.9 ± 3.9	100.8 ± 3.9	101.1 ± 3.7
CYP3A4(M) *	93.2 ± 13.2	97.8 ± 4.3	96.2 ± 4.3	96.8 ± 4.2	96.9 ± 7.2	96.7 ± 6.2	97.9 ± 4.4	99.7 ± 4.1
CYP3A4(T) *	95.3 ± 2.9	98.5 ± 4.2	95.1 ± 3.5	98.0 ± 2.4	95.3 ± 1.7	90.8 ± 3.4	95.9 ± 4.2	95.2 ± 0.5

* CYP3A4(M)-1-OH-midazolam; CYP3A4(T)–6-β-OH-Testosterone.

**Table 3:** Effects of GCS on CYP-specific metabolite formation in human liver microsomes (*n* = 3).

CYP Isozyme	Remaining Activities (% of Control, *n* = 3)							
	GCS (μM)							
	0.3	1	3	10	30	100	300	1000
CYP1A2	110.2 ± 20.5	103.1 ± 1.5	101.1 ± 7.5	94.2 ± 1.8	94.1 ± 3.0	97.2 ± 3.7	86.7 ± 2.8	82.5 ± 12.0
CYP2A6	107.6 ± 5.2	105.3 ± 0.4	106.2 ± 2.3	101.5 ± 1.4	102.1 ± 2.5	103.4 ± 2.7	97.9 ± 0.4	94.1 ± 7.3
CYP2B6	121.0 ± 11.6	116.2 ± 5.8	114.3 ± 3.5	108.2 ± 3.1	107.8 ± 1.3	111.2 ± 2.3	105.6 ± 1.8	103.5 ± 9.2
CYP2C9	115.5 ± 15.2	108.7 ± 1.7	109.2 ± 2.4	104.0 ± 2.3	105.8 ± 2.7	101.2 ± 4.8	97.2 ± 2.8	91.1 ± 10.1
CYP2C19	128.4 ± 27.9	102.5 ± 3.0	102.6 ± 8.8	101.9 ± 4.3	90.5 ± 8.7	100.3 ± 3.7	95.5 ± 5.6	88.7 ± 7.3
CYP2D6	107.6 ± 7.4	104.9 ± 1.0	106.2 ± 3.7	101.6 ± 0.5	100.4 ± 1.4	102.6 ± 1.7	100.6 ± 1.3	99.5 ± 4.8
CYP3A4(M) *	104.4 ± 13.5	103.5 ± 3.2	98.3 ± 1.9	97.2 ± 1.5	96.9 ± 6.1	95.2 ± 8.2	91.0 ± 3.3	88.7 ± 12.1
CYP3A4(T) *	92.8 ± 0.6	92.4 ± 0.9	91.1 ± 3.1	91.7 ± 4.4	91.8 ± 3.0	84.8 ± 7.8	82.1 ± 3.4	80.0 ± 6.6

* CYP3A4(M)-1-OH-midazolam; CYP3A4(T)–6-β-OH-Testosterone.

**Table 4:** List of drugs used for OA, metabolism-associated CYPs, and CYP-mediated drug interactions for selected analgesics and NSAIDs from guidelines.

Drug Type	Drug	Metabolism-Associated CYPs	CYP-Mediated Drug Interactions	Refs
Analgesics	Acetaminophen	CYP3A4 (major), CYP2El, CYP1A2, CYP2D6	Isoniazid (CYP2E1 inducer) Carbamazepine (CYP3A4 inducer) Rifampicin (CYPs inducer)	[17,18]
	Tramadol	CYP2D6 (major), CYP3A4, CYP2B6	Terbinafine (CYP2D6 inhibitor) Escitalopram (CYP2D6 inhibitor) Rifampicin (CYPs inducer)	[19,20,21,24]
	Codeine	CYP3A4, CYP2D6 (major), CYP2C8	Bupropion (CYP2D6 inhibitor) Paroxetine (CYP2D6 inhibitor) Fluoxetine (CYP2D6 inhibitor)	[22,25]
	Pethidine	CYP2B6 (major), CYP3A4 (major), CYP2C19	Phenobarbitone (CYP inducer) Chlorpromazine (CYP inhibitor)	[23,26]
NSAIDs	Ibuprofen	CYP2C8 (major) CYP2C9 (major), CYP2C19, CYP3A4	Voriconazole (CYP2C9 inhibitor) Fluconazole (CYP2C9 inhibitor) Gemfibrozil (CYP2C8 inhibitor)	[27,28,29]
	Diclofenac	CYP2C8, CYP2C9 (major), CYP2C18, CYP2C19, CYP2B6, CYP3A4	Voriconazole (CYP2C9 inhibitor)	[30,31]
	Meloxicam	CYP2C9 (major), CYP3A4	Amiodarone (CYP2C9 inhibitor) Voriconazole (CYP2C9 inhibitor) Itraconazole (CYP3A4 inhibitor)	[32,33,34]
	Celecoxib	CYP2C9 (major), CYP3A4	Fluconazole (CYP2C9 inhibitor) Fluvastatin (CYP2C9 inhibitor) Rifampicin (CYP2C9 inducer)	[35,36]

## Data Availability

The data presented in this study are available in article or Supplementary Material.

## Supplementary Materials

The following supporting information can be downloaded at: https://www.mdpi.com/article/10.3390/molecules28248068/s1. Table S1. The guideline recommendations for the most commonly used oral and topical pharmacological agents in OA treatment. Table S2. Linearity data of probe metabolites (*n* = 3). Table S3. Accuracy and precision data of probe metabolites intra-day, inter-day for LC-MS/MS validation. Table S4. Precursor-product ion pairs of CYP-specific metabolites for multiple reaction monitoring. Figure S1. Calibration curves of probe metabolites. Figure S2. Chromatograms data of probe metabolites for selectivity. Blank, control, spiked sample of medium quality control.
