# Supplementary material for: Investigation of Drug-Interaction Potential for Arthritis Dietary Supplements: Chondroitin Sulfate, Glucosamine, and Methylsulfonylmethane
Source: Molecules. 2023 Dec 13;28(24):8068. doi: 10.3390/molecules28248068 (PMC10745882; doi:10.3390/molecules28248068)
Supplement: Supplementary file 1 [file molecules-28-08068-s001.zip › molecules-2725154-supplementary.pdf]

Investigation of Drug-Interaction Potential for Arthritis Dietary Supplements: Chondroitin Sulfate, Glucosamine, and Methylsulfonylmethane

Su Min Kim [1,†], So Young Jo [1,†], Ho-Young Park [2], Yu Ra Lee [2], Jun Sang Yu [1,*] and Hye Hyun Yoo [1,*]

[1] Institute of Pharmaceutical Science and Technology, College of Pharmacy, Hanyang University,
Ansan 15588, Republic of Korea; ksm990201@naver.com (S.M.K.); sjo7909@gmail.com (S.Y.J.)

[2] Food Functionality Research Division, Korea Food Research Institute,
Wanju-gun 55365, Republic of Korea; hypark@kfri.re.kr (H.-Y.P.); lyr@kfri.re.kr (Y.R.L.)

* Correspondence: xat007@hanyang.ac.kr (J.S.Y.); yoohh@hanyang.ac.kr (H.H.Y.);
Tel.: +82-31-400-4783 (J.S.Y.); +82-31-400-5804 (H.H.Y.)

† These authors contributed equally to this work.

Supporting Information

Table S1. The guideline recommendations for the most commonly used oral and topical pharmacological agents in OA treatment [1].

Guideline Author	Year	Acetaminophen	Opioid Analgesics		Peroral NSAIDs	SYSADOA	Topical NSAIDs
			Tramadol	Other			
AAOS	2013	Unable to give any recommendation	Positive recommendation	Inconclusive	Positive recommendation	Strong recommendation against use	Positive recommendation
ACR/AF	2020	Conditional recommendation for	Conditional Recommendation for	Conditional recommendation against	Recommended as first-line treatment	Strong recommendation against use	Strong recommendation for use prior to oral NSAIDs
OARSI	2019	Conditional recommendation for	Strong recommendation against		Recommended as first-line treatment	Not included	Recommended as first-line treatment
ESCEO	2019	Weak recommendation against as single therapy, should be used as rescue medicine in addition to first-line treatment with SYSADOA	Conditional recommendation for as third-line treatment		Recommended as first-line, short-term treatment	Recommended as first-line, long-term treatment for pharmaceutical-grade products	Recommended in addition to SYSADOA and acetaminophen prior to oral NSAIDs

Note: AAOS-American Academy of Orthopedic Surgeons; ACR/AF-American College of Rheumatolo-gy/Arthritis Foundation; OARSI-Osteoarthritis Research Society International; ESCEO-European Society for Clinical and Economic Aspects of Osteoporosis, Osteoarthritis and Musculoskeletal Diseases;

Table S2. Linearity data of probe metabolites (n=3).

Metabolites	Concentration (%)											
	2.0		5.0		10.0		20.0		50.0		100.0	
	Accuracy (%)	RSD (%)	Accuracy (%)	RSD (%)	Accuracy (%)	RSD (%)	Accuracy (%)	RSD (%)	Accuracy (%)	RSD (%)	Accuracy (%)	RSD (%)
Acetaminophen	96.48	2.26	100.80	2.82	99.32	1.73	100.70	0.92	104.30	4.28	98.03	0.45
7-OH-coumarin	100.33	6.67	103.56	4.74	92.71	2.51	94.66	3.28	102.87	2.84	105.86	7.05
OH-bupropion	101.05	2.06	99.31	3.84	98.76	0.13	94.30	1.91	95.63	0.60	110.94	11.20
Dextrorphan	99.89	1.97	100.98	1.41	98.91	1.97	94.29	2.57	114.57	3.32	90.86	1.26
4-OH-diclofenac	101.95	5.31	96.87	5.84	97.21	1.33	97.73	1.28	97.98	2.92	108.26	9.24
4-OH-mephenytoin	101.52	11.62	96.18	1.66	101.39	2.83	95.84	3.39	102.21	3.00	102.85	7.39
1-OH-midazolam	98.44	3.90	105.08	13.14	99.32	1.11	97.12	3.51	97.67	1.26	102.38	4.70
6-β-OH-Testosterone	100.26	1.50	99.17	4.00	102.21	4.82	96.55	5.24	96.72	0.22	105.09	7.16

Table S3. Accuracy and precision data of probe metabolites intra-day, inter-day for LC-MS/MS validation.

Metabolites	Intra-day (n=3)						Inter-day (3days, n=3/day)					
	Accuracy (%)			RSD (%)			Accuracy (%)			RSD (%)		
	LQC	MQC	HQC	LQC	MQC	HQC	LQC	MQC	HQC	LQC	MQC	HQC
Acetaminophen	103.49	96.20	100.77	2.98	0.84	0.72	98.44	93.52	97.70	4.31	3.56	0.33
7-OH-coumarin	105.02	96.05	94.60	4.19	1.83	3.63	104.29	97.75	91.10	3.02	1.54	0.79
OH-bupropion	100.47	99.45	103.53	3.57	0.41	1.77	100.97	100.09	102.51	0.56	1.28	0.96
Dextrorphan	90.24	104.97	88.12	0.79	1.76	0.49	95.01	101.70	90.30	0.85	4.13	0.07
4-OH-diclofenac	100.77	96.69	100.25	2.83	0.71	0.46	102.12	99.68	98.16	1.09	0.84	1.15
4-OH-mephenytoin	100.50	88.47	90.11	8.26	1.87	1.37	101.86	89.22	96.56	2.35	0.60	2.62
1-OH-midazolam	98.90	96.34	93.70	0.07	1.77	1.86	101.48	100.37	91.74	2.79	4.13	0.28
6-β-OH-Testosterone	106.15	95.93	93.62	10.98	3.69	1.95	103..74	97.03	92.47	2.35	3.35	1.37

LQC-low quality control; MQC-medium quality control; HQC-high quality control.

Table S4. Precursor-product ion pairs of CYP-specific metabolites for multiple reaction monitoring.

P450 isozyme	Metabolites monitored	Precursor ion	Product ion
CYP 1A2	Acetaminophen	152.1	110.1
CYP 2A6	7-OH-coumarin	162.9	106.9
CYP 2B6	OH-bupropion	256.2	238.2
CYP 2D6	Dextrorphan	258.2	157.1
CYP 2C9	4-OH-diclofenac	312.2	230.9
CYP 2C19	4-OH-mephenytoin	235.0	150.1
CYP 3A4	1-OH-midazolam	343.1	325.1
CYP 3A4	6-β-OH-testosterone	305.2	269.2

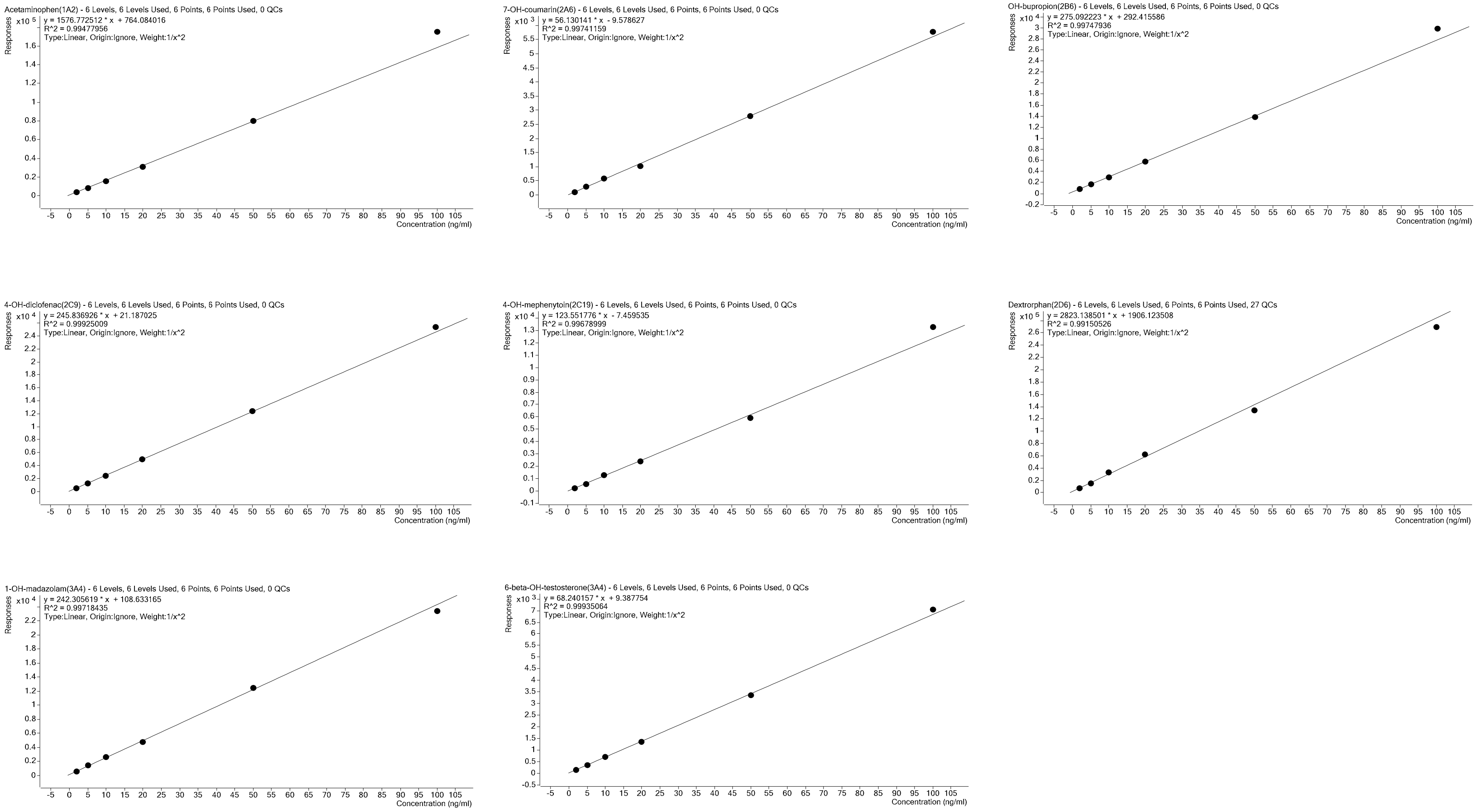

Figure S1. Calibration curves of probe metabolites.

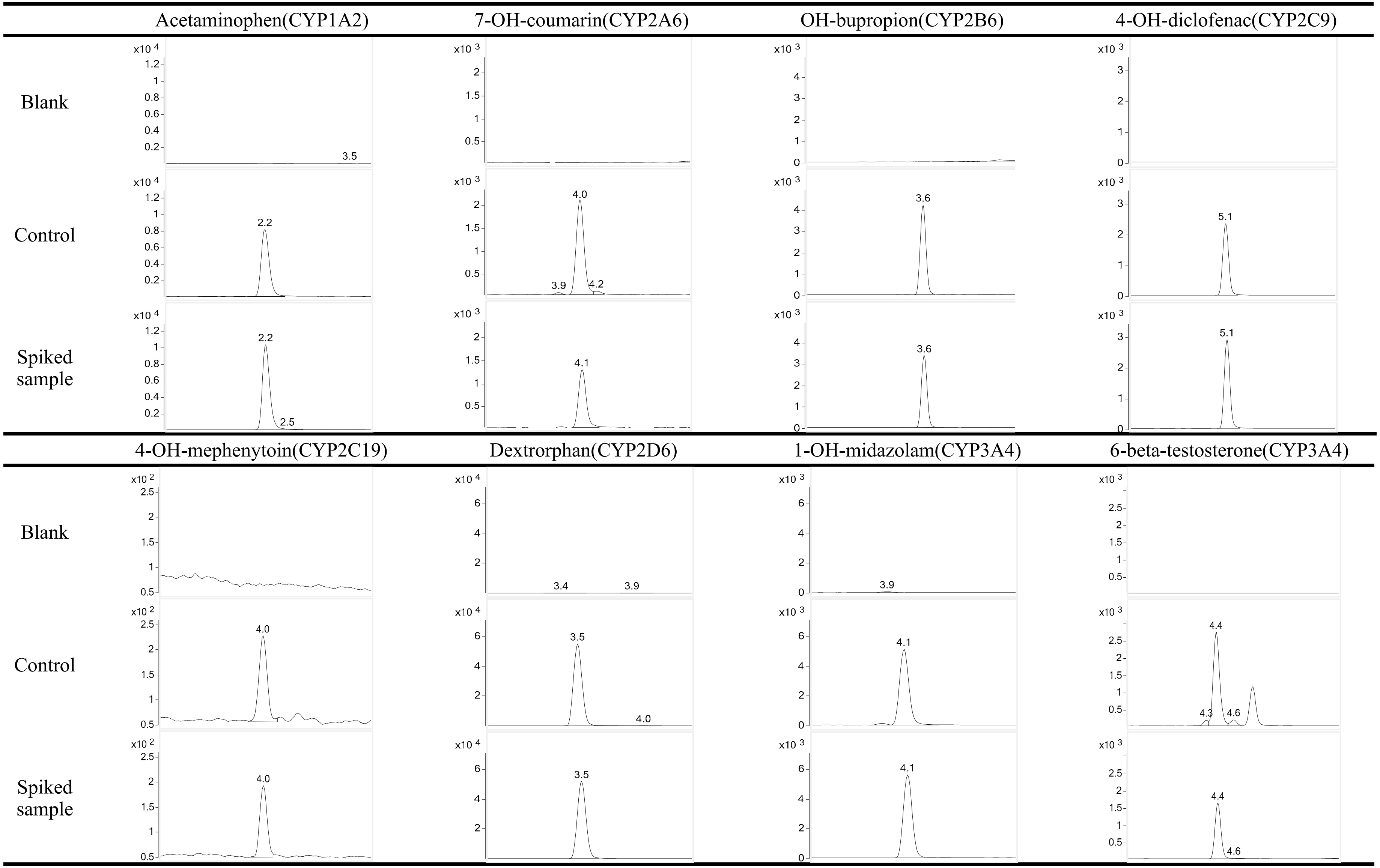

Figure S2. Chromatograms data of probe metabolites for selectivity. Blank, control, spiked sample of medium quality control.

References

1. Primorac, D.; Molnar, V.; Matisic, V.; Hudetz, D.; Jelec, Z.; Rod, E.; Cukelj, F.; Vidovic, D.; Vrdoljak, T.; Dobricic, B.; Anticevic, D.; Smolic, M.; Miskulin, M.; Cacic, D.; Boric, I., Comprehensive Review of Knee Osteoarthritis Pharmacological Treatment and the Latest Professional Societies' Guidelines. *Pharmaceuticals-Base* **2021,** 14, (3).
